# Unveiling the Viral Landscape in Gastric Cancer

**DOI:** 10.1007/s00284-026-05120-9

**Published:** 2026-08-19

**Authors:** Diego Pereira, Fabiano Cordeiro Moreira, Daniel de Souza Avelar, Sérgio Augusto Antunes Ramos, Valéria Cristiane Santos da Silva, Jéssica Manoelli Costa da Silva, Ronald Matheus da Silva Mourão, Rubem Ferreira da Silva, Kauê Sant’Ana Pereira Guimarães, Juliana Barreto Albuquerque Pinto, Marcos da Conceição, Williams Fernandes Barra, Samia Demachki, Samir Mansour Casseb, Rommel Mario Rodriguez Burbano, Paulo Pimentel de Assumpção

**Affiliations:** 1 Postgraduate Program in Genetics and Molecular Biology, Federal University of Pará, Belém, Pará Brazil; 2 Oncology Research Center, Federal University of Pará, Belém, Pará Brazil; 3 Postgraduate Program in Biotechnology, Federal University of Pará, Belém, Pará Brazil; 4 Postgraduate Program in Oncology and Medical Sciences, Federal University of Pará, Belém, Pará Brazil; 5 Undergraduate Program in Biotechnology, Federal University of Pará, Belém, Pará Brazil; 6 Molecular Biology Laboratory, Ophir Loyola Hospital, Belém, Pará Brazil

## Abstract

The present study aimed to comprehensively characterize the cancer-associated virome in patients with gastric cancer. A total of 452 samples were analyzed, including 226 tumor tissues and 202 peritumoral tissues from patients undergoing surgical resection, alongside 24 normal gastric mucosa samples obtained via endoscopy from individuals without cancer. RNA was extracted, libraries were prepared, and paired-end sequencing was performed on the NextSeq 500 platform (Illumina, US). High-quality reads were processed with Kraken2, using the standard database supplemented with the Reference Viral Database, to identify viral taxa. Taxonomic profiles, differential viral abundance, and alpha and beta diversity metrics were computed in RStudio, with statistical analyses performed to compare viral composition and diversity among tissue groups using Kruskal-Wallis, Wilcoxon, PERMANOVA, and PERMDISP tests (*p* < 0.05). Across all gastric tissues, 47 viral orders and 637 viral genera were detected, of which only 10.6% were shared among tissue types, suggesting low conservation and instability of the gastric virome. Gastric tumors exhibited high relative abundance of bacteriophages and clinically relevant eukaryotic viruses, along with reduced viral richness and diversity and marked heterogeneity among tumors. Overall, this study reveals tissue-specific and heterogeneous viral communities, with tumors showing reduced diversity among rare taxa while dominant viruses remain stable, highlighting ecological instability, patient-specific virome reorganization, and the potential of the gastric virome as a biomarker and target for tumor–virome studies.

## Introduction

 Gastric cancer (GC) is a malignant neoplasm of the gastrointestinal tract and ranks as the fifth most common cancer worldwide [1]. Although incidence and mortality rates have declined in recent years, GC still exhibits a high mortality rate, reaching approximately 75% in many countries [2]. The low rate of early diagnosis results in most patients being identified at advanced stages of the disease, thereby missing the optimal window for clinical intervention. Under these circumstances, the prognosis remains poor, with a five-year post-surgical survival rate below 30% [3, 4].

GC is a highly heterogeneous neoplasm, characterized by multiple distinct phenotypes, with most cases being gastric adenocarcinomas (GAC). Although often considered a single entity, GC can be classified into two categories based on anatomical location: cardia gastric cancer (upper stomach) and non-cardia gastric cancer (lower stomach) [5]. The main risk factors for cardia GC include obesity and gastroesophageal reflux disease, whereas chronic *Helicobacter pylori* infection is considered the primary cause of non-cardia GC [6, 7].

In recent decades, several studies have investigated the mechanisms of gastric carcinogenesis using sequencing technologies, with a focus on the relationship between gastric microbiome and GC development. Recent evidence suggests that microorganisms may play a significant role both in tumorigenesis and in cancer therapeutic strategies [8]. Moreover, the application of high-throughput sequencing technologies in microbiology has revealed that the stomach, previously considered an inhospitable environment for microorganisms, harbors a diverse and functional microbiota [9].

The mechanisms by which the gastric microbiome contributes to carcinogenesis are not yet fully understood. However, it is recognized that a dysbiotic microbiota can increase the risk of GC through the production of microbial metabolites capable of inducing chronic inflammation in gastric tissue. This inflammatory process promotes the accumulation of mutations and genomic instability in the host, accelerating DNA replication rates and impairing DNA damage repair mechanisms [10, 11]. Moreover, these alterations may be further amplified by interactions between the gastric microbiota, *H. pylori*, and the host immune responses [12–14].

The human virome is predominantly composed of eukaryotic viruses and bacteriophages, whose dysbiosis has been associated with a variety of diseases, including cancer [15]. Among eukaryotic viruses, oncoviruses are particularly notable for their well-established oncogenic mechanisms. Conversely, bacteriophages can indirectly influence carcinogenesis by modulating the composition and virulence of the bacterial microbiota [16]. Despite advances in characterizing the bacterial component of the gastric tumor microbiome and its association with carcinogenesis, the viral component remains poorly explored, mainly due to technical challenges in detecting viral material and limitations of existing databases [17].

In this study, we revealed a highly heterogeneous and tissue-specific gastric virome, with distinct viral signatures associated with tumoral, peritumoral, and normal tissues. The observed alterations in viral diversity and composition highlight the potential role of the virome as an unexplored component of the gastric tumor microenvironment and a source of candidate biomarkers for gastric cancer.

## Materials and Methods

### Sample Collection and Total RNA Extraction

A total of 452 samples were analyzed, comprising 226 tumor tissues (GAC) and 202 peritumoral tissues (PTT) collected from patients with gastric adenocarcinoma undergoing surgical resection. Additionally, 24 normal gastric mucosa samples (NT) were used as controls and were obtained by upper gastrointestinal endoscopy from individuals without gastric adenocarcinoma. From each specimen, approximately 30 mg of tissue was macerated, and RNA was extracted using the TRIzol Reagent method (Thermo Fisher Scientific) in accordance with the manufacturer’s protocol. The integrity and concentration (ng/µL) of the extracted RNA were evaluated using a Qubit 4 Fluorometer (Thermo Fisher Scientific). Only samples with an RNA Integrity Number (RIN) ≥ 5 were considered acceptable.

### Library Construction and RNA Sequencing

RNA libraries were prepared using the TruSeq Stranded Total RNA Library Prep Kit with Ribo-Zero Gold (Illumina, US) following the manufacturer’s protocol, with approximately 1 µg of total RNA per sample in a final volume of 11 µL. Library quality was evaluated using the 2200 TapeStation System (Agilent Technologies, Basel, Switzerland). Sequencing was carried out in paired-end mode on the NextSeq 500 platform (Illumina, US) with the NextSeq 500 MID Output V2 kit (150 cycles), according to the manufacturer’s instructions.

### Sequence Data Pre-processing

Post-sequencing, raw RNA-Seq reads underwent quality control. Read quality was initially assessed using FastQC, followed by adapter trimming and filtering of low-quality reads using FASTP (v1.0.1) applying a quality score threshold of quality value > 15.

### Taxonomic Classification

For viral identification, high quality and clean reads were processed with Kraken2 (v2.1.6), employing the standard database supplemented with the Reference Viral Database (RVDB) (v28.0) [18] to improve viral detection. The Kraken2 outputs were processed in RStudio software (v2024.04.2) using the pavian package (v1.2.1).

### Taxonomic Profiling and Differential Abundance Analysis

The output files were further processed in RStudio using the phyloseq (v1.54.0) and microbiome (v1.32.0) packages to calculate relative abundances at the order and genus taxonomic levels. Shared and unique viral genera between groups were assessed and visualized using a Venn diagram with the ggvenn package (v0.1.19) and an UpSet plot generated with the UpSetR package (v1.4.0). Statistically significant differences in the abundance of taxa among groups were identified through the linear discriminant analysis effect size (LEfSe) algorithm, as implemented in the microbiomeMarker R package (v1.13.2). Only taxa with an LDA score greater than 3.0 and a p-value < 0.05 (Wilcoxon or Kruskal–Wallis test) were considered significantly enriched. Bar plots of relative abundance analyses and LEfSe were generated using the ggplot2 package (v4.0.1), while heatmaps were constructed with the ComplexHeatmap package (v2.26.0).

### Diversity Analysis

Alpha diversity was assessed across groups using the phyloseq package (v1.54.0) by calculating Observed richness, Chao1, Shannon, and Simpson indices. Statistical differences in alpha diversity between groups were evaluated using Mann–Whitney (Wilcoxon) tests. Beta diversity was evaluated using the vegan package (v2.7.2) based on Bray–Curtis dissimilarity and the Jaccard index to assess differences in viral community composition among samples. Principal Coordinates Analysis (PCoA) was performed for both distance metrics to visualize compositional patterns. Multivariate differences in community structure were tested using non-parametric permutational multivariate analysis of variance (PERMANOVA), implemented through the adonis function. Homogeneity of multivariate dispersions among groups was assessed using PERMDISP based on both Bray–Curtis and Jaccard distances, with statistical significance evaluated by permutation testing (999 permutations). All plots were generated with the ggplot2 package (v4.0.1). Statistical significance was defined as a p-value < 0.05.

## Results

### Taxonomic Virome Profiles Differ Among Tumoral, Peritumoral, and Normal Gastric Tissues

**Fig. 1:**
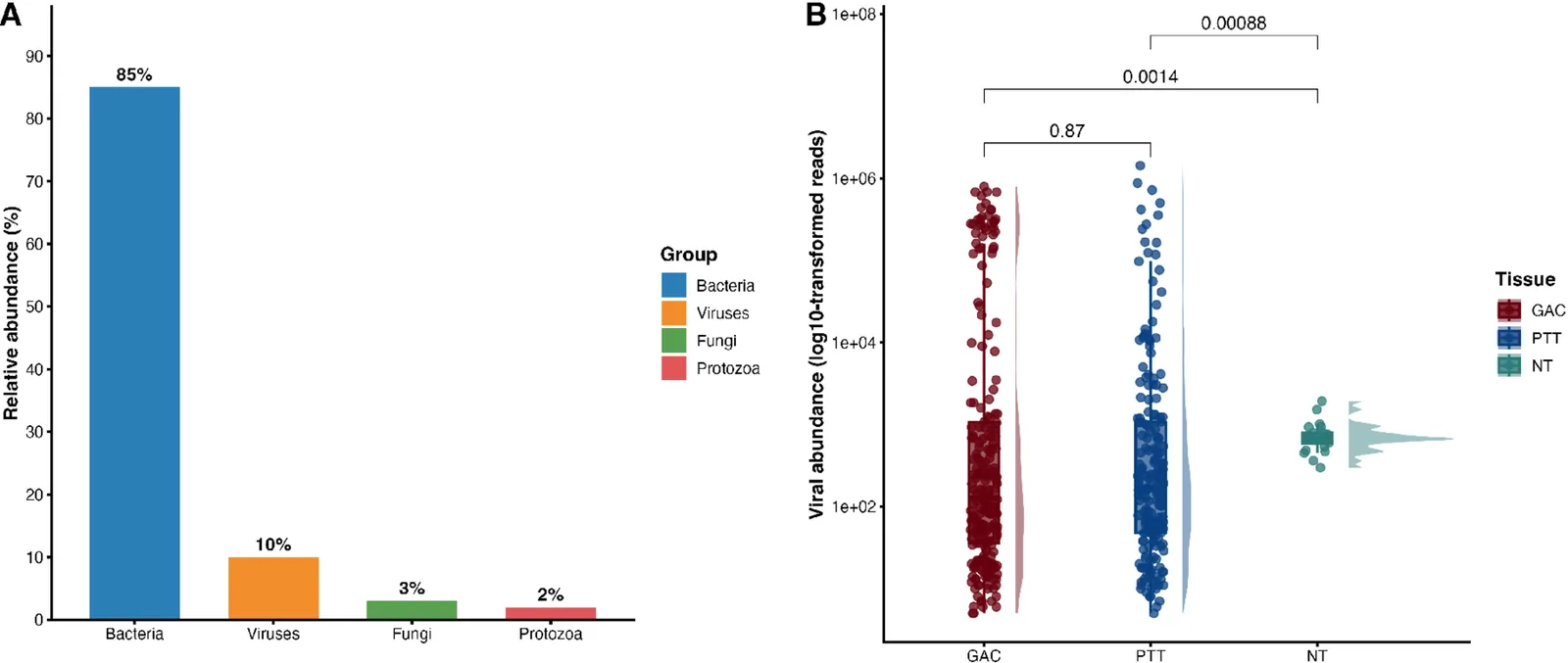
Overview of microbial composition across gastric tissues. (**a**) Relative contribution of microbial groups to the overall sequencing dataset. (**b**) Viral read abundance across gastric tissue types. Statistical comparisons were performed using the Wilcoxon test, with significance defined as *p* < 0.05

In the present study, RNA-Seq was employed to investigate the virome in gastric adenocarcinoma, enabling a comprehensive assessment of viral diversity and composition across distinct gastric tissue groups. The microbial taxonomic profiles of 452 gastric samples, comprising 226 gastric adenocarcinoma (GAC), 202 peritumoral (PTT), and 24 normal gastric mucosa (NT) samples, were characterized using the Kraken2 algorithm. Microbial reads were detected in all samples and classified into bacterial, viral, fungal, and protozoan taxa, with only viral taxa retained for downstream analyses (Fig. 1A). Overall, 1,754,380 viral reads were identified across all samples, corresponding to an average of approximately 3,881 viral reads per sample. Most viral reads originated from GAC samples (66.41%), followed by PTT samples (32.11%), whereas NT samples accounted for only 1.48% of the total viral reads (Fig. 1B).

**Fig. 2:**
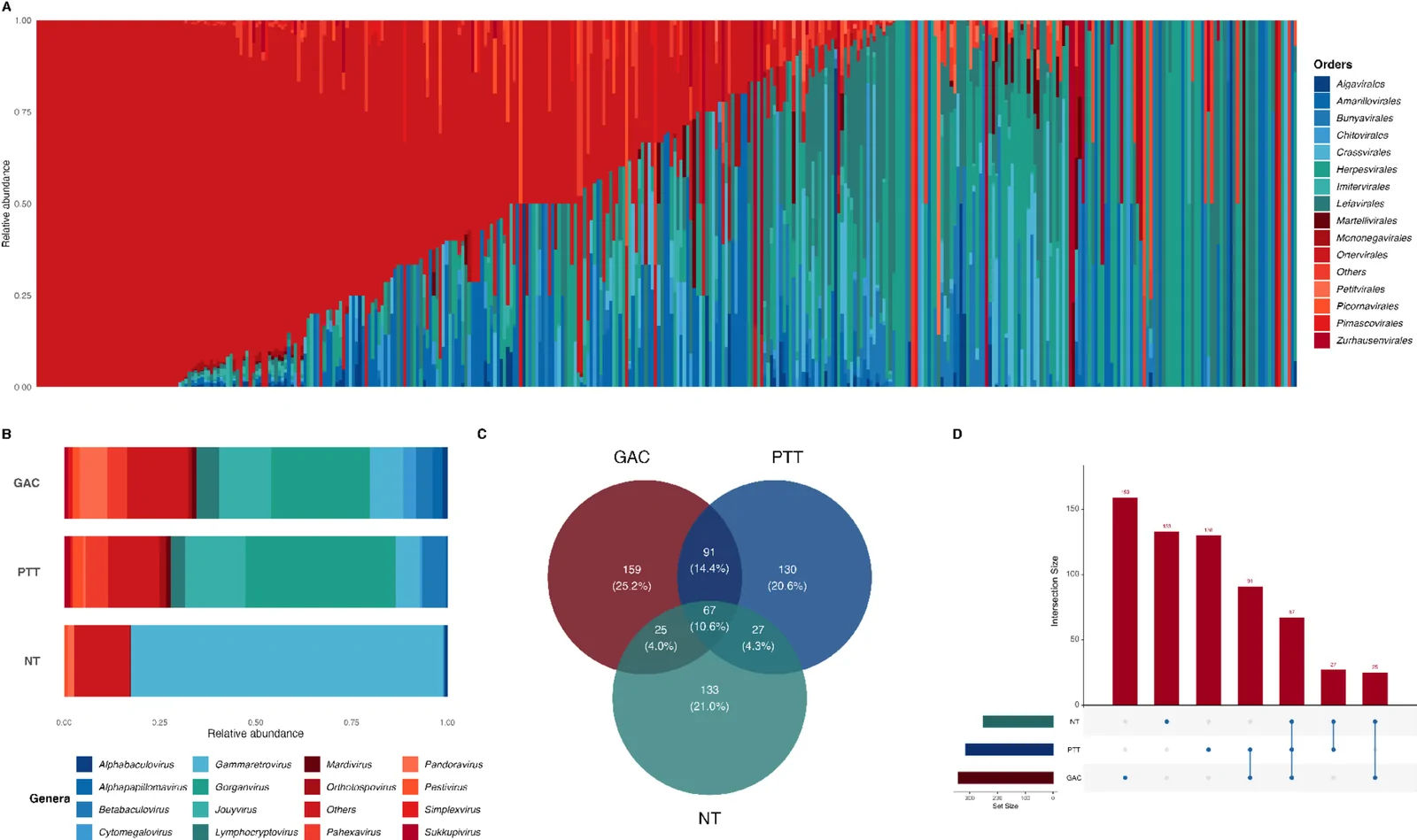
Taxonomic profile of the gastric virome. (**a**) Relative abundance of the most abundant viral orders across all samples. (**b**) Relative abundance of the most abundant viral genera across all samples. (**c**) Venn diagram showing the overlap of the virome profiles among gastric tissue types at the genus level. (**d**) UpSet plot of the core virome profiles among gastric tissue types at the genus level

The viral composition was subsequently examined in more detail at both the order and genus levels. At the order level, a total of 47 viral orders were identified across the entire dataset, with *Ortervirales*, *Herpesvirales*, *Amarillovirales*, *Zurhausenvirales* and *Picornavirales* representing the most abundant orders overall (Fig. 2A). At the genus level, a total of 637 unique genera were identified across all samples analyzed. The relative abundance of the top 15 genera overall is shown in Fig. 2B. In GAC, *Gorganvirus*,* Jouyvirus*, *Gammaretrovirus*, *Pandoravirus* and *Lymphocryptovirus* exhibited the highest relative abundances. PTT were predominantly characterized by *Gorganvirus*,* Jouyvirus*,* Gammaretrovirus*,* Betabaculovirus*, and *Pahexavirus*. In NT, the most abundant genera included *Gammaretrovirus*,* Pegunavirus*,* Pandoravirus*,* Pestivirus*, and *Alphacrustrhavirus.* The 15 most abundant viral genera in each group, ordered in decreasing abundance, are presented in Table 1. Of the 637 viral genera detected across all samples, a substantial proportion was tissue-specific, with 25.2% (159/637) genera uniquely associated with GAC, 20.6% (130/637) exclusive to PTT, and 21% (133/637) restricted to NT. In contrast, only 10.6% (67/637) of the virome composition in each group were shared, suggesting that the gastric virome is relatively unstable and shows low conservation across gastric tissue types (Fig. 2C-D).

**Table 1:** The top 15 most abundant viral genera among gastric tissue types

Order	GAC	PTT	NT
1	*Gorganvirus*	*Gorganvirus*	*Gammaretrovirus*
2	*Jouyvirus*	*Jouyvirus*	*Pegunavirus*
3	*Gammaretrovirus*	*Gammaretrovirus*	*Pandoravirus*
4	*Pandoravirus*	*Betabaculovirus*	*Pestivirus*
5	*Lymphocryptovirus*	*Pahexavirus*	*Alphacrustrhavirus*
6	*Pahexavirus*	*Lymphocryptovirus*	*Alphabaculovirus*
7	*Betabaculovirus*	*Pestivirus*	*Prasinovirus*
8	*Cytomegalovirus*	*Orthotospovirus*	*Luchadorvirus*
9	*Alphapapillomavirus*	*Sukkupivirus*	*Backyardiganvirus*
10	*Pestivirus*	*Mardivirus*	*Jeilongvirus*
11	*Alphabaculovirus*	*Moineauvirus*	*Betabaculovirus*
12	*Mardivirus*	*Simplexvirus*	*Megavirus*
13	*Sukkupivirus*	*Pandoravirus*	*Crinivirus*
14	*Simplexvirus*	*Cytomegalovirus*	*Mollyvirus*
15	*Orthotospovirus*	*Alphatorquevirus*	*Friunavirus*

GAC – Gastric adenocarcinoma; PTT – Peritumoral tissue; NT – Normal tissue

### Specific Viral Genera Exhibit Tissue-Specific Enrichment Among Gastric Tissues

**Fig. 3:**
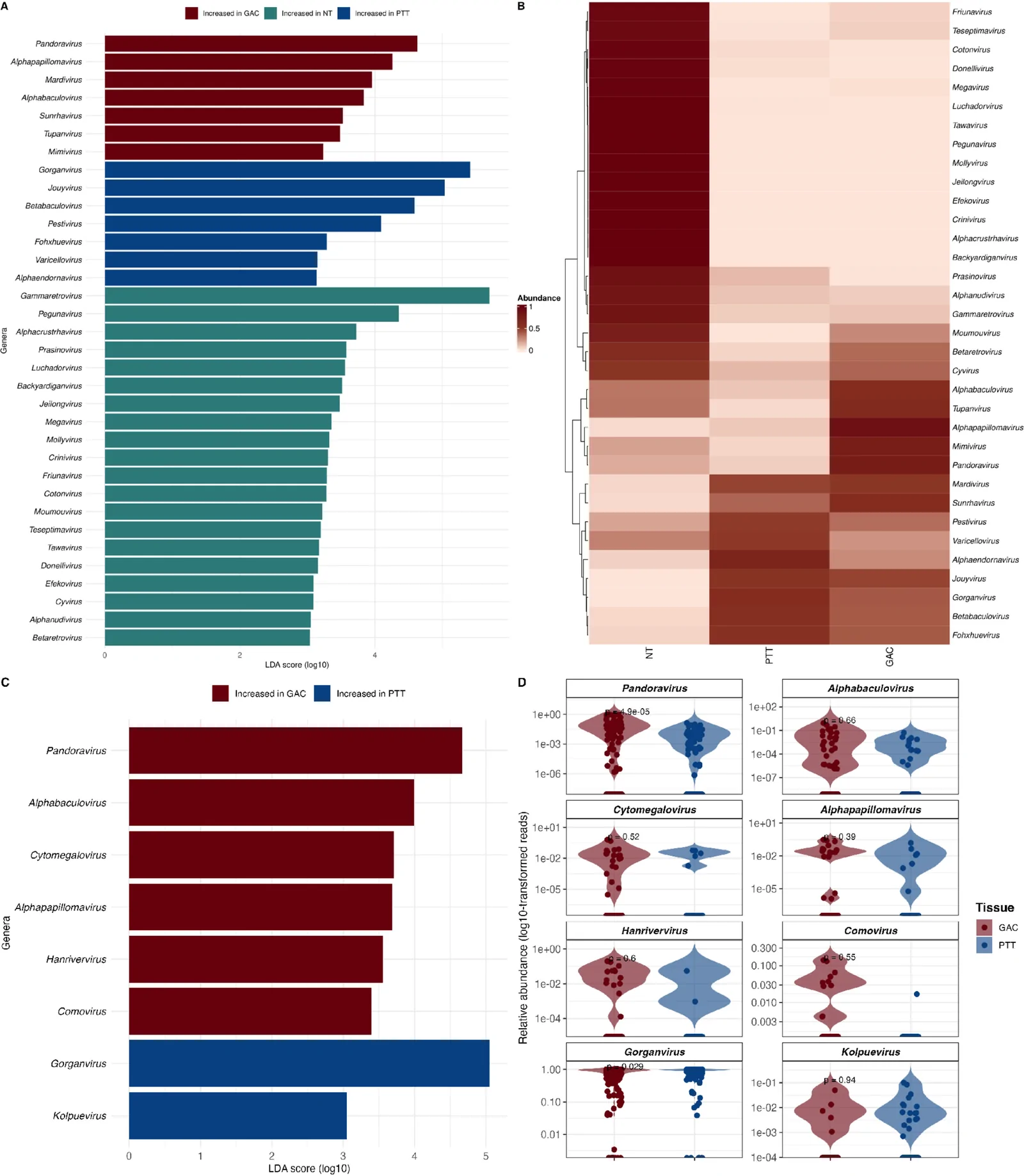
Differential viral abundance among gastric tissue types. (**a**) Association of specific viral genera with gastric tissue types by LEfSe. (**b**) Heatmap showing tissue-specific enrichment patterns of viral genera identified by LEfSe. (**c**) Association of differentially abundant viral genera between GAC and PTT tissues by LEfSe. (**d**) Boxplots showing the relative abundance of viral genera differentially enriched between GAC and PTT tissues. Viral genera with an LDA score > 3.0 were considered significantly enriched. Statistical comparisons were performed using the Kruskal-Wallis test and the Wilcoxon rank-sum test, with significance defined as *p* < 0.05

To identify the most relevant taxa driving the differences among groups, a LEfSe analysis was performed. This analysis identified 34 viral genera that were differentially abundant among groups (Fig. 3A). GAC samples were primarily characterized by an enrichment of viral taxa belonging to the genera *Pandoravirus*,* Alphapapillomavirus*,* Mardivirus*,* Alphabaculovirus*, and *Sunrhavirus*. In contrast, *Gorganvirus*,* Jouyvirus*,* Betabaculovirus*,* Pestivirus*, and *Fohxhuevirus* were the genera that most strongly discriminated the virome of PTT. With respect to NT, the genera *Gammaretrovirus*,* Pegunavirus*,* Alphacrustrhavirus*,* Luchadorvirus*, and *Prasinovirus* were the most significantly associated with this tissue type. To further explore the abundance patterns of the viral genera identified by the LEfSe analysis, a heatmap was generated including only the differentially enriched taxa (Fig. 3B). This analysis revealed distinct and consistent tissue-specific profiles, with several genera displaying marked enrichment in a single tissue type and reduced representation in the others. Overall, the differential enrichment of viral genera across tissue types highlights a structured and tissue-dependent organization of the gastric virome. To further investigate the viral differences between tumor and peritumor tissues, a differential abundance analysis was performed comparing GAC and PTT samples directly. This analysis identified *Pandoravirus*,* Alphabaculovirus*,* Cytomegalovirus*,* Alphapapillomavirus*,* Hanrivervirus*, and *Comovirus* as significantly enriched in GAC, whereas *Gorganvirus* and *Kolpuevirus* were significantly enriched in PTT (Fig. 3C-D). These findings indicate that, despite sharing several viral taxa, the tumor and peritumoral tissues exhibit distinct viral signatures, supporting the presence of virome remodeling within the tumor-associated microenvironment.

### Gastric Adenocarcinoma is Associated with Reduced Viral Alpha Diversity

**Fig. 4:**
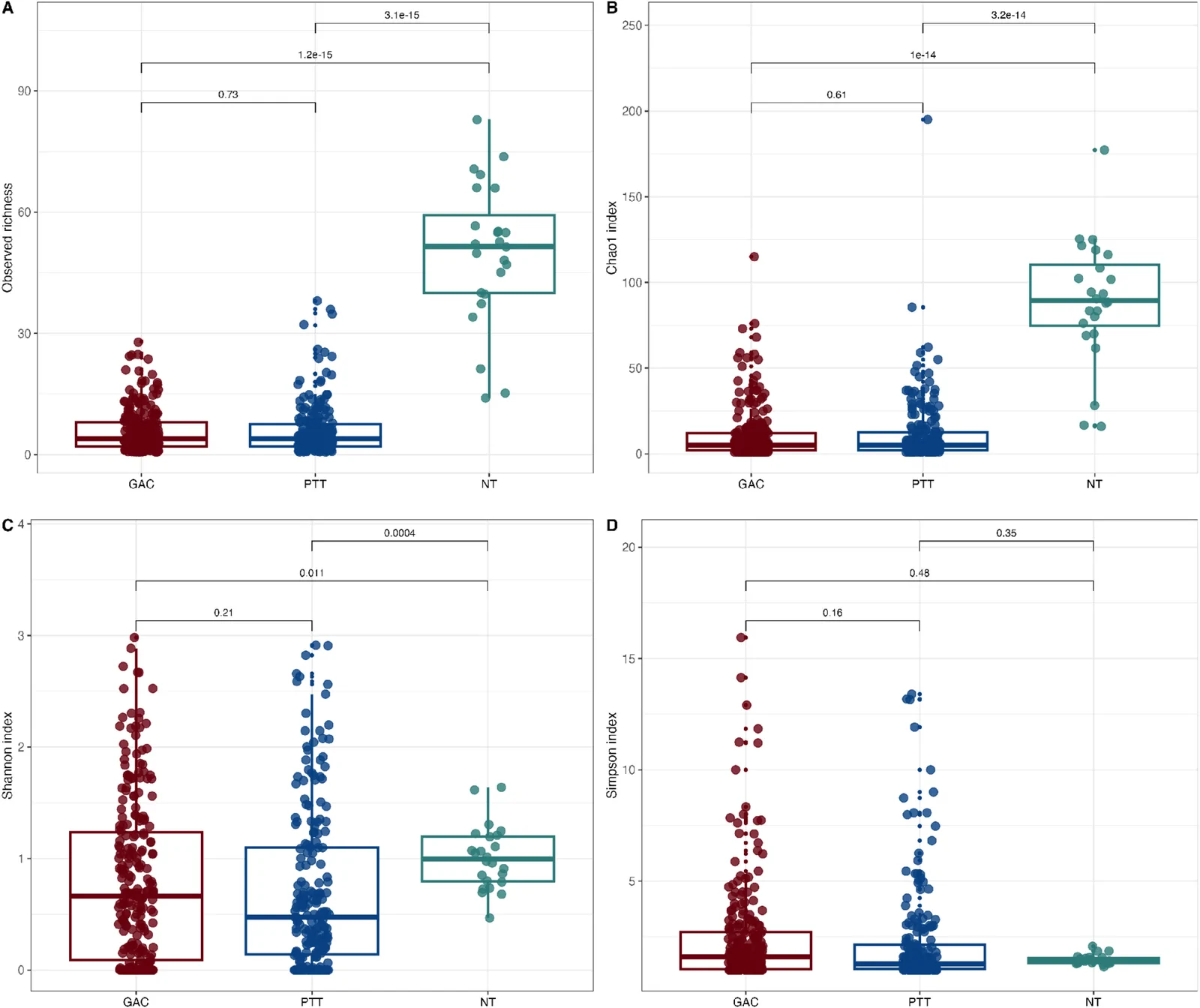
Viral alpha diversity among gastric tissue types. (**a**) Observed richness. (**b**) Chao1 index. (**c**) Shannon index. (**d**) Simpson index. Statistical comparisons were performed using the Wilcoxon test, with significance defined as *p* < 0.05

Alpha diversity quantifies the variety of taxa within a sample, considering both richness and evenness in the distribution of individuals. Alpha diversity of the gastric virome was assessed using observed richness, Chao1, and the Shannon and Simpson indices, revealing distinct patterns among the analyzed groups. Based on observed richness, NT exhibited significantly higher viral richness compared with both GAC (*p* = 1.2 × 10⁻¹⁵) and PTT (*p* = 3.1 × 10⁻¹⁵), whereas no significant difference was observed between GAC and PTT (*p* = 0.73) (Fig. 4A). Similarly, based on the Chao1 index, NT also showed significantly higher viral richness compared with GAC (*p* = 1.0 × 10⁻¹⁴) and PTT (*p* = 3.2 × 10⁻¹⁴), with no significant difference detected between GAC and PTT (*p* = 0.61) (Fig. 4B). Consistently, Shannon diversity was significantly higher in NT than in GAC (*p* = 0.011) and PTT (*p* = 0.0004), while no statistically significant difference was detected between GAC and PTT (*p* = 0.21) (Fig. 4C). In contrast, the Simpson index did not differ significantly among groups, including comparisons between GAC and PTT (*p* = 0.16), GAC and NT (*p* = 0.48), and PTT and NT (*p* = 0.35) (Fig. 4D). Collectively, these results indicate that gastric adenocarcinoma and the associated peritumoral tissue are characterized by a significant reduction in viral alpha diversity, primarily driven by losses in richness and evenness, whereas the dominance structure of the viral community remains relatively preserved.

### Beta Diversity Reveals High Intra-Group Heterogeneity in the Gastric Adenocarcinoma

**Fig. 5:**
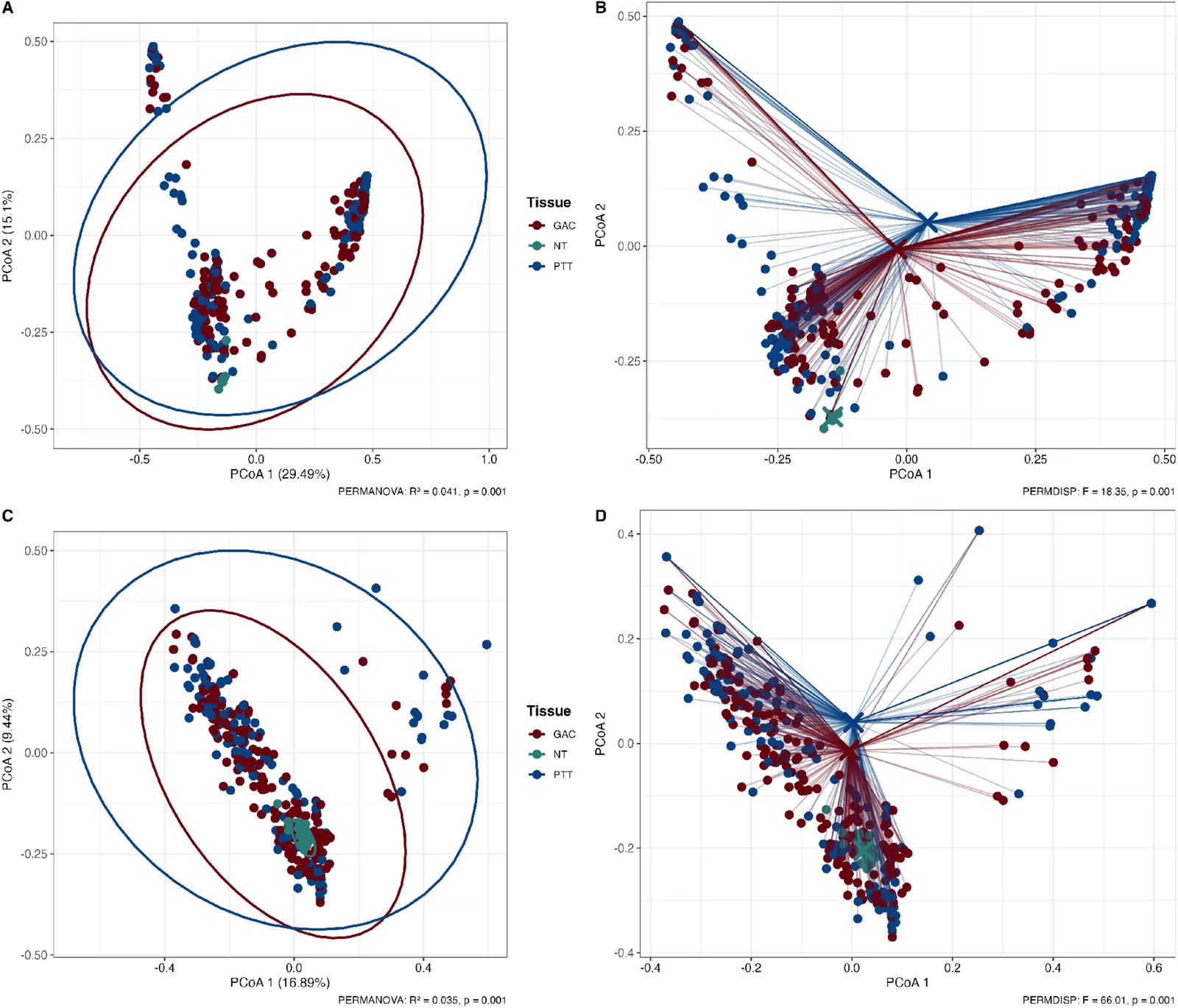
Viral beta diversity among gastric tissue types. (**a**) PCoA based on Bray–Curtis dissimilarity. (**b**) Bray–Curtis-based multivariate dispersion. (**c**) PCoA based on the Jaccard index. (**d**) Jaccard-based multivariate dispersion. Statistical comparisons were performed using the PERMANOVA and PERMDISP, with significance defined as *p* < 0.05

Beta diversity quantifies differences in taxonomic composition between samples or communities, allowing the assessment of similarity or dissimilarity among groups. To assess differences in viral community composition among groups, beta diversity was evaluated using both Bray–Curtis dissimilarity, which accounts for relative abundances, and the Jaccard index, which considers presence/absence of viruses. PCoA based on Bray–Curtis distances showed partial separation among groups, with the first three axes explaining 29.5%, 15.1%, and 10.8% of the total variation, respectively (Fig. 5A). In agreement with this pattern, PERMANOVA revealed a statistically significant difference in community composition between groups (*p* = 0.001), although the effect size was small (R² = 0.041), indicating that most variation occurs within groups. Assessment of the homogeneity of multivariate dispersions using PERMDISP revealed significant differences in dispersion among groups (F = 18.35, *p* = 0.001), with tumoral and peritumoral tissues showing greater dispersion of samples around their centroids, whereas normal tissues exhibited lower dispersion, indicating more homogeneous community composition within this group (Fig. 5B). Similarly, PCoA based on Jaccard distances revealed differences in viral presence/absence patterns among groups, with the first three axes explaining 16.9%, 9.4%, and 5.6% of the total variation, respectively (Fig. 5C). PERMANOVA again indicated statistically significant differences among groups (*p* = 0.001), despite a low effect size (R² = 0.035), suggesting limited separation between groups relative to within-group variability. Consistently, PERMDISP detected significant differences in dispersion among groups (F = 66.01, *p* = 0.001), indicating that the observed heterogeneity in within-group viral composition was driven by tumoral and peritumoral tissues, which exhibited significantly greater dispersion of samples around their centroids compared with normal tissue, based on presence/absence data (Fig. 5D).

## Discussion

Currently, several studies have investigated the role of microorganisms in the initiation and progression of gastric cancer. However, beyond *Helicobacter pylori*, the specific contribution of other components of the microbiota to gastric carcinogenesis has not been clearly established. Historically, gastric tissue was considered sterile due to its continuous exposure to a highly acidic environment. However, with the advancement of sequencing technologies, accumulating evidence has demonstrated that the stomach harbors a diverse microbial community [9, 19].

In the present study, a total of 47 viral orders were detected in the analyzed gastric tissue samples. Among the most abundant, *Ortervirales*, *Herpesvirales*, *Zurhausenvirales* and *Picornavirales*, stand out, as they include viruses capable of infecting a wide range of vertebrate hosts, including humans [20–23]. *Crassvirales* and *Petitvirales* comprise major families of viruses that infect bacteria [24, 25]. At the genus level, a total of 637 viral genera were detected across the gastric tissue samples. Of these, 159 were exclusive to tumoral tissues, 130 to peritumoral tissues, and 133 to normal gastric mucosa. Interestingly, only 10.6% (67/637) were shared among gastric tissue types. This pattern underscores the largely tissue-specific composition of the gastric virome, revealing pronounced heterogeneity in the distribution of viral genera among tumoral, peritumoral, and normal gastric tissues.

In gastric peritumoral tissues, the genera *Gorganvirus*,* Jouyvirus*,* Kolpuevirus*, and *Fohxhuevirus* were found to be differentially abundant. These viruses are characterized by their specificity for bacterial hosts and are classified as bacteriophages. While *Gorganvirus* and *Jouyvirus* have been described as infecting *Proteus spp*. and *Escherichia coli*, respectively, specific bacterial hosts have not yet been identified for *Kolpuevirus* and *Fohxhuevirus* [26–29]. Several of these bacterial hosts have been associated with gastrointestinal dysbiosis and inflammatory processes, suggesting that the enrichment of their corresponding bacteriophages may represent an indirect signature of microbial community shifts within the gastric tumor-associated microenvironment, particularly the altered microbial landscape surrounding the tumor, rather than an isolated alteration of the virome [30].

Although these bacteriophages belong to distinct viral genera, they share common biological characteristics, including a double-stranded DNA (dsDNA) genome and a strict dependence on bacterial hosts for replication [31]. This functional convergence suggests that their enrichment across the gastric tumor microenvironment, encompassing both tumor and peritumoral tissues, may represent a shared ecological signature associated with microbiota remodeling rather than merely reflecting viral taxonomic diversity. By influencing bacterial community structure, horizontal gene transfer, bacterial fitness, and virulence traits, these phages may contribute to the establishment of a dysbiotic microbial landscape and to the inflammatory conditions that characterize the gastric tumor microenvironment. Thus, alterations in bacteriophage communities may represent an additional layer of microbial ecosystem disruption potentially involved in gastric carcinogenesis and tumor progression [32].

Eukaryotic viruses with tropism for human cells can establish latent or persistent infections, modulate host immune responses, and, in some cases, contribute to acute or chronic diseases [15]. In the present study, the genera *Lymphocryptovirus*,* Cytomegalovirus*, and *Alphapapillomavirus* represented the most prevalent eukaryotic viruses associated with human cells detected in gastric tumors. The identification of these clinically relevant viral groups in gastric tumor tissues is noteworthy, given their established roles in human infections and their ability to persist within host cells. These viruses share biological features, including double-stranded DNA (dsDNA) genomes and the capacity to establish long-term interactions with host cells [15]. Notably, although these viruses possess DNA genomes, they were identified through metatranscriptomic RNA sequencing, indicating the presence of viral transcripts within the tumor tissue. This finding suggests ongoing viral transcriptional activity, which may be consistent with active infection or transcriptionally active latent infection. Consequently, their presence in the gastric tumor microenvironment may reflect sustained virus–host interactions capable of modulating immune responses, promoting persistent inflammatory signaling, and altering tissue homeostasis. Together, these mechanisms have been implicated in tumor development and progression, including gastric carcinogenesis [33].

*Lymphocryptovirus* was detected exclusively in tumoral and peritumoral gastric tissues in this study. Although its relative abundance did not differ significantly between these two tissue types, its presence in gastric tumors may have direct implications for carcinogenesis. *Lymphocryptovirus humangamma4*, commonly known as Epstein–Barr virus (EBV), is the primary human representative of this genus and is well established as an oncogenic virus, being associated with several hematological malignancies, including Burkitt and Hodgkin lymphomas, as well as with epithelial cancers such as nasopharyngeal and gastric carcinomas [34–36]. EBV-associated gastric tumors account for approximately 9% of gastric adenocarcinomas and represent a distinct subtype in terms of oncogenesis and molecular features, highlighting the importance of detecting this virus in gastric tumors [37]. EBV-related gastric carcinogenesis is primarily mediated by the expression of viral proteins, microRNAs (BART), and the BARF1 protein, which promote malignant transformation through the induction of methylation and the regulation of host gene expression [38].

Another relevant eukaryotic virus differentially abundant in gastric tissues was *Cytomegalovirus*. The representative species associated with human infection is *Cytomegalovirus humanbeta5*, commonly known as human cytomegalovirus (HCMV), one of the eight human herpesviruses. HCMV is characterized by its ability to establish lifelong latency in the host and to undergo periodic reactivation [39]. Although not formally classified as an oncogenic virus, accumulating evidence suggests that HCMV may contribute to cancer development and progression by acting as an initiator or promoter of carcinogenesis, with reported associations in breast and colorectal cancers [40, 41]. Recent evidence suggests that *Cytomegalovirus* infection may play a role in the progression or development of gastric cancer, potentially through modulation of the tumor microenvironment, particularly in immunocompromised patients [42].

*Alphapapillomavirus* infections, commonly referred to as Human papillomaviruses (HPVs), are highly prevalent among sexually active adults [43]. HPV types 16 and 18 are the most common and are strongly linked to viral oncogenesis. The malignancies associated with these types primarily include cervical, penile, vulvar, vaginal, anal, and oropharyngeal carcinomas [44]. In the present study, *Alphapapillomavirus* were significantly enriched in gastric adenocarcinoma. Previous investigations have reported the presence of high-risk human papillomaviruses (HPVs) in gastric carcinomas, including a study from Iran that detected HPV in 5% (5/100) of cases [45] and another analysis identifying HPV sequences in 10 out of 361 gastric tumor samples, of which five were genotyped as high-risk HPV-16. Notably, active expression of the E6 and E7 oncogenes was observed in only one of these cases [46]. Although HPVs are classically associated with epithelial infections of the skin and oral or genital mucosae, their detection in gastric tissues has been repeatedly reported, and their potential contribution to gastric carcinogenesis remains unclear.

Both gastric tumor and peritumoral tissues exhibited differential abundance of viral genera associated with non-human hosts. *Pestivirus* species are commonly identified in mammals such as cattle [47], whereas *Mardivirus*, a member of the herpesvirus family, predominantly infects domestic birds [48]. The detection of these viruses in human gastric tissues is unlikely to indicate active infection and may instead reflect transient exposure, dietary-derived viral sequences, residual nucleic acids from ingested material, or environmental contamination. Similarly, other differentially abundant genera, including *Varicellovirus*, *Betabaculovirus*, and *Alphaendornavirus*, lack established associations with the gastrointestinal tumor microenvironment. Therefore, the presence of these viral signatures should be interpreted with caution, as they may represent exogenous viral material or technical limitations in taxonomic classification rather than biologically relevant infections within gastric tissues.

Gastric tumor and peritumoral tissues exhibited a consistent reduction in viral diversity compared with normal gastric tissue, characterized by lower richness and decreased diversity metrics integrating richness and evenness, as demonstrated by alpha diversity analyses. No substantial differences were observed between tumoral and peritumoral tissues, indicating that both compartments share a similar pattern of viral community alteration. Additionally, the overall dominance structure of the viral community remained relatively stable across groups, suggesting that tumor-associated changes extend beyond the tumor core and influence the surrounding peritumoral tissue. These findings indicate that alterations within the gastric tumor microenvironment primarily affect low-abundance and rare viral taxa, leading to reduced viral diversity while preserving the relative dominance of the remaining viral populations.

Beta diversity analyses revealed modest but consistent differences in viral community composition among gastric tissue types, with partial separation between groups observed using both abundance-based and presence/absence-based metrics. In both approaches, variation within groups exceeded variation between groups, and a pronounced heterogeneity was evident in tumoral and peritumoral tissues, whereas normal gastric tissue displayed a more homogeneous viral community structure. This pattern suggests that tumor-associated microenvironmental changes do not impose a uniform reshaping of the virome but instead generate diverse and patient-specific viral configurations. Intrinsic alterations in the tumor-associated microenvironment may create heterogeneous niches that selectively favor or restrict different viral taxa across tumoral and peritumoral regions. In contrast, the reduced dispersion observed in normal tissue is consistent with a more stable and regulated gastric environment, supporting a conserved viral community structure. Together, these findings indicate that gastric tumor development is associated less with a directional shift in viral composition and more with increased ecological instability and heterogeneity of the virome.

## Conclusions

This study provides a comprehensive characterization of the gastric virome, revealing a highly tissue-specific and heterogeneous viral landscape. Gastric tumors harbor distinct viral genera, including eukaryotic viruses with human tropism. Some viral genera exhibited tissue-specific enrichment, highlighting that certain viruses preferentially associate with tumoral, peritumoral, or normal gastric tissues. Tumoral and peritumoral tissues exhibited a marked reduction in viral diversity, primarily affecting low-abundance and rare taxa, whereas the relative dominance of prevalent viral populations remained largely unchanged. Beta diversity analyses further highlighted pronounced heterogeneity in tumoral and peritumoral tissues, in contrast to the more homogeneous viral communities observed in normal gastric mucosa. Collectively, these findings suggest that gastric tumor development is associated with increased ecological instability and patient-specific reorganization of the virome, rather than a uniform directional shift, and underscore the potential of the gastric virome as a valuable, yet largely unexplored, component of the tumor microenvironment with implications for biomarker discovery and understanding host-microbe interactions in cancer.

## Data Availability

The datasets generated during and/or analysed during the current study are available from the corresponding author on reasonable request.
